# Adverse Reactions to Dry Needling Therapy: Insights from Polish Physiotherapy Practice

**DOI:** 10.3390/jcm13237032

**Published:** 2024-11-21

**Authors:** Robert Trybulski, Adrian Kużdżał, Marek Kiljański, Kamil Gałęziok, Filip Matuszczyk, Adam Kawczyński, Filipe Manuel Clemente

**Affiliations:** 1 Medical Department Wojciech Korfanty Upper Silesian Academy, September Scouts Street 3, 40-659 Katowice, Poland; 2 Provita Żory Medical Center, 44-240 Żory, Poland; 3 Institute of Health Sciences, College of Medical Sciences, University of Rzeszów, 35-310 Rzeszów, Poland; kuzdzal.a@gmail.com; 4 PAZARE Medical Center Pabianice, Bracka 54, 95-200 Pabianice, Poland; 5 Department of Biomechanics and Sport Engineering, Gdansk University of Physical Education and Sport, 80-336 Gdansk, Poland; kawczynski.a@gmail.com; 6 Escola Superior de Desporto e Lazer, Instituto Politécnico de Viana do Castelo, Rua Escola Industrial e Comercial de Nun’Álvares, 4900-347 Viana do Castelo, Portugal; 7 Research & Innovation Center in Sport Physical Activity, and Health, 4900-347 Viana do Castelo, Portugal

**Keywords:** dry needling, physiotherapy, therapeutics, adverse effects

## Abstract

**Background/Objectives**: The current study aimed to characterize the adverse reactions associated with dry needling (DN) treatments reported by Polish physiotherapists, with a secondary objective of investigating whether the level of DN experience influences the occurrence of these adverse effects. **Methods**: A total of 102 Polish physiotherapists, all with regular DN practice, participated in an ad hoc online survey. The participants were categorized by their level of experience in DN treatment: 44 with 0–2 years, 43 with 3–6 years, and 15 with more than 7 years of experience. The survey consisted of 27 questions focused on both mild and severe adverse effects resulting from DN treatments. **Results**: The results showed that in the short term (over the past week), minor bleeding was the most commonly reported mild adverse effect (70%), followed closely by slightly pain during treatment (68%). Slight pain following treatment was also reported by 52% of respondents. No significant relationship was found between experience with dry needling (DN) and the reporting of mild adverse effects, with the exception of tingling (X_(2)_ = 10.958; *p* = 0.004). In the retrospective analysis of the past month, most respondents reported experiencing bleeding between one and three times (49%), while bruising occurred one to three times in 44% of cases. Similarly, 44% of respondents noted pain after treatment one to three times, and 47% experienced pain during DN at this frequency. A significant interaction with DN experience was observed in the frequency of drowsiness reported over the past month (X_(10)_ = 19.735; *p* = 0.032). **Conclusions**: Severe adverse effects were extremely rare in clinical practice: pneumothorax and shock were each reported by 3% of respondents, nerve palsy by 14%, infection by 2%, and hospitalization by 1%. In conclusion, this study suggests that most adverse effects are mild, typically involving bleeding and slight pain during or after treatment. Additionally, DN experience does not appear to be a significant factor influencing the type or prevalence of these adverse effects.

## 1. Introduction

Dry needling (DN) is a therapeutic technique used in physiotherapy involving the insertion of fine, solid needles into myofascial trigger points—tight bands or knots within a muscle that cause pain and restrict movement [1]. Unlike acupuncture, which is rooted in traditional Chinese medicine, DN is based on Western anatomical and neurophysiological principles [2]. Its primary aims are to release muscle tension, decrease pain, and restore function by disrupting dysfunctional neuromuscular junctions and stimulating blood flow [3]. Evidence shows that DN can effectively relieve pain in conditions like chronic lower back pain [4], neck pain [5], shoulder pain [6], and even tension-type headaches [7]. DN is particularly beneficial in treating musculoskeletal conditions and myofascial pain syndromes by reducing local inflammation and enhancing the body’s natural healing processes [8].

Common techniques include superficial DN, where the needle is inserted just below the skin, and deep DN, where the needle penetrates deeper to target the muscle directly at the trigger point [9]. Clinicians may use a “pistoning” technique, rapidly inserting and withdrawing the needle to stimulate the muscle or provoke a “local twitch response”—an involuntary muscle contraction believed to disrupt tight muscle bands and decrease pain [10]. Another approach is “static needling”, where the needle remains in the muscle for a period to allow for sustained tissue stimulation [2]. Although DN is generally considered safe, adverse effects can occur in response to the technique, depending on factors such as needle placement, depth, or angle [11]. Additionally, improper technique or pre-existing health conditions, such as infections or nerve sensitivity, can increase the risk of side effects like bruising, soreness, or more serious complications [12]. These are unexpected and undesirable events directly associated with the care or DN services provided to a patient.

A previous study [13] covering 20,464 DN treatment sessions indicated that 36.7% of patients experienced mild adverse effects, with the most common being bleeding, bruising, and pain during the procedure. In another survey conducted in Ireland, 19.18% of participants reported experiencing mild adverse effects, with bruising, bleeding, and pain during treatment being the most commonly reported [14]. In Australia, a survey of physiotherapists regarding adverse effects also revealed that the most commonly reported mild adverse effects were discomfort during treatment (62%) and bruising (56%) [15]. Severe complications are rare but can happen, especially if needling is performed in sensitive areas or close to critical structures [1]. These include infections, nerve or blood vessel injury, and, in very rare cases, pneumothorax (collapsed lung) if the needle is inserted too deeply into the chest area [16].

Monitoring adverse effects in DN is crucial for physiotherapists to ensure patient safety and optimize treatment outcomes [17]. Close monitoring allows clinicians to quickly identify and manage any complications, helping prevent mild issues from escalating into serious conditions. Moreover, the experience level of physiotherapists may potentially influence the risk of adverse effects, as a previous study observed that being male, being experienced (>4 years), and having more training were associated with a major adverse event occurring during a physiotherapist’s career [18]. Consequently, both thorough training and ongoing vigilance are essential in minimizing potential harm and delivering effective care in DN.

Despite existing evidence on the adverse effects of DN, there is a lack of analysis in Poland, and no data currently support the establishment of a standardized approach to reporting these adverse events. It is important not only to characterize how DN is being implemented in physiotherapy practices but also to identify the main mild and severe adverse effects associated with DN, in order to inform and regulate clinical practice policies in the country. Furthermore, comparing different levels of experience with DN could provide valuable insights into whether a certain threshold of experience influences the frequency or severity of adverse effects. For these reasons, a cross-sectional online survey was conducted to characterize the adverse reactions associated with DN treatments reported by Polish physiotherapists, with a secondary objective of examining whether the level of DN experience affects the occurrence of these adverse effects.

## 2. Materials and Methods

Since this study was approved by the National Council of Physiotherapists (ID: 26/2022; Approved: 12 January 2023), this cross-sectional observational anonymous survey was conducted. In accordance with the principles outlined in the Declaration of Helsinki, this study adhered to ethical standards for research involving human subjects. Informed consent was obtained from all participants prior to their involvement in the survey. It is important to highlight that participation was voluntary, with physiotherapists having the option to withdraw from this study at any time without facing any penalties or adverse consequences.

### 2.1. Study Design

Aiming to analyze the adverse effects of DN practices, this study employed a cross-sectional design and utilized an online survey targeting physiotherapists.

### 2.2. Study Setting

Between January and March 2024, physical therapist members of the Polish Society of Manual Medicine members and the Polish Society of Physiotherapy were surveyed. These specific practitioners were targeted due to their Polish-based membership and the likelihood of including physical therapists trained in DN. This survey was conducted electronically through Google forms, a web-based platform for research surveys. The Polish Society of Physiotherapy distributed the survey link via electronic newsletters or membership-wide emails. This survey was designed to be entirely anonymous in order to prioritize confidentiality and encourage honest responses. This approach allowed participants to provide their feedback and insights freely, ensuring a secure and confidential environment that promoted openness and trust.

### 2.3. Participants

Eligible participants were physical therapists who received the survey link, irrespective of whether they practiced dry needling. The key inclusion criteria were being a certified physical therapist and using dry needling treatments in their practice, regardless of whether they held formal dry needling certification. Exclusion criteria involved participants who left questions regarding their years of experience in physiotherapy practice and their use of dry needling unanswered, as both are essential for determining eligibility based on the ability to perform DN.

Out of a total of 109 respondents, 102 met the eligibility criteria and were included in the data analysis. The main demographic information of the participants is presented in Table 1.

### 2.4. Variables

In this study, the number of years of experience with DN was used as an independent factor to compare the occurrence of mild and severe adverse effects across different experience levels. Participants were classified as novices if they had 0–2 years of DN practice, intermediate if they had 3–6 years, and experts if they had more than 7 years of experience with DN.

### 2.5. Data Sources/Measurement

To investigate the adverse effects of DN, an ad hoc survey was developed for this study. Initially drafted by a team of three researchers, this survey focused on key concepts, including mild and severe adverse DN effects, with questions based on the existing literature [13,18].

To ensure the quality and validity of the survey, a draft was reviewed by three independent academic experts in DN, all recognized DN researchers according to ExpertScape (https://expertscape.com/ex/dry+needling) accessed on 20 October 2024. Their feedback was instrumental in refining the survey, leading to revisions in both the wording and structure of the questions.

Subsequently, the revised version was sent to a group of three additional practitioner experts, including four Polish physiotherapists with at least 15 years of being licensed and certified in DN. Their insights contributed to further enhancing the content of the survey and ensuring cultural and language validity.

Following the incorporation of feedback from both the academic experts and physiotherapists, the final version of the survey was returned to the original three academic experts for their final review. After receiving their approval, the survey was made available as Supplementary File S1 and launched online.

Before proceeding with the questionnaire, participants were given a clear explanation of the survey’s purpose, research design, and assurances regarding anonymity, confidentiality, and data protection. Their consent and voluntary participation were confirmed prior to completing the survey.

The survey included a demographic section with 6 closed-ended questions designed to collect data on participants’ academic and professional backgrounds and clinical practice. There were 12 questions on mild adverse effects (any unintended and non-therapeutic ill effects, short-term and non-serious, with no impact on function) to assess the frequency of occurrence in the week prior to the survey and in the month prior. Regarding severe adverse effects (any unintended and non-therapeutic ill effects that are serious, distressing, and may necessitate additional treatment), 5 questions were included. Finally, there were 4 questions regarding the characteristics of clinical practices and interaction with patients.

### 2.6. Study Size

The recommended a priori sample size of 77 was calculated using G*power software (version 3.1) for a chi-squared goodness-of-fit test (contingency table option). This calculation was made with a *p*-value of 0.05, a power of 0.85, and an effect size threshold of 0.4, corresponding to a small effect size. The choice of this effect size was due to the absence of similar studies that could provide the mean difference between populations. Additionally, a degrees of freedom value of 3 was assigned, as most studies involved dichotomous questions comparing three groups of practitioners based on experience levels.

### 2.7. Quantitative Variables and Statistical Methods

The data obtained from the survey were first exported and organized into an Excel file for further analysis. For questions with fixed responses, a frequency analysis was carried out to evaluate the distribution and occurrence of various response options. This enabled a quantitative analysis of participants’ selections within the defined categories.

To analyze potential associations between two categorical variables, such as the years of experience with DN and survey responses, a chi-squared test was used. These statistical procedures were executed using SPSS software (version 28.0.0.0, IBM, Chicago, IL, USA), with a significance level set at a *p*-value of <0.05.

## 3. Results

Table 1 provides general demographic information about the physiotherapists who participated in this survey. Among the respondents (n = 102), the majority were men (69%), with most participants falling in the age range of 26–35 years. The largest portion of respondents had 3–6 years (25%) and 7–10 years (21%) of experience practicing physical therapy. Almost all participants were certified in the dry needling (DN) technique (96%). Regarding DN-specific experience, most respondents had 0–2 years (n = 44) or 3–6 years (n = 43) of experience, while a smaller group reported having over 7 years of experience in DN (n = 15). In terms of weekly DN practice, the majority performed the technique 1–10 times per week (55%). No significant interactions were found between experience in DN and the frequency with which the physical therapist performs DN each week (X_(12_) = 14.775; *p* = 0.254).

Table 2 presents the cases of mild adverse effects reported in the past week related to the use of DN treatment. Minor bleeding was the most reported effect (70%), followed closely by slight pain during treatment (68%). Slight pain following treatment was also noted by 52% of respondents. Less common effects included headache (2%), nausea (3%), tingling (7%), and the exacerbation of primary pain symptoms (7%). Interestingly, significant interactions between DN experience and mild adverse effects were observed in weekly reports of tingling (X_(2)_ = 10.958; *p* = 0.004). Tingling was reported more frequently by PTs with over 7 years of experience (27%) compared to those with 0–2 years (5%) or 3–6 years (2%) of experience.

No significant interactions were found between experience in DN and the weekly frequency of reports on slight pain during DN treatment (X_(2)_ = 0.495; *p* = 0.781), slight pain after DN treatment (X_(2)_ = 4.421; *p* = 0.110), feeling weakness (X_(2)_ = 2.779; *p* = 0.249), minor bleeding (X_(2)_ = 4.782; *p* = 0.092), bruising (X_(2)_ = 5.263; *p* = 0.072), nausea (X_(2)_ = 0.855; *p* = 0.652), drowsiness (X_(2)_ = 4.651; *p* = 0.098), the exacerbation of the primary pain symptoms (X_(2)_ = 0.672; *p* = 0.715), and headaches (X_(2)_ = 2.799; *p* = 0.247).

Table 3 presents adverse effects reported over the past month in DN practice. Bleeding was reported to occur between one and three times by the majority of respondents (49%), and bruising also frequently occurred one to three times (44%). Pain after treatment was similarly reported to occur one to three times by 44% of respondents, and pain during DN was reported at this frequency by 47%. Most respondents reported no occurrence of primary symptom exacerbation (75%), drowsiness (67%), weakness (67%), headache (88%), or nausea (86%). No significant interactions were found between experience in DN and the last month’s frequency of reports on bleeding after DN treatment (X_(14)_ = 16.383; *p* = 0.291), bruising (X_(10)_ = 7.031; *p* = 0.723), pain after treatment (X_(12)_ = 11.711; *p* = 0.469), pain during treatment (X_(12)_ = 13.207; *p* = 0.354), exacerbation of primary pain symptoms (X_(4)_ = 7.576; *p* = 0.108), feeling weakness (X_(10)_ = 17.598; *p* = 0.062), headaches (X_(4)_ = 6.802; *p* = 0.147), and nausea (X_(6)_ = 4.367; *p* = 0.627). However, significant interactions were observed in the frequency of drowsiness reported over the past month (X_(10)_ = 19.735; *p* = 0.032), with 27% of the more experienced respondents (i.e., >7 years of practice) reporting 7 to 15 occurrences in the previous month.

Table 4 shows the typical pain levels reported by patients during DN procedures. During treatment, most patients reported pain scores of 1–2 (29%) and 3–4 (26%) on the numeric pain rating scale. After treatment, the majority reported lower pain levels, with a score of 1–2 (46%). No significant interactions were found between experience in DN and the numeric pain rating scale scores reported during DN treatment (X_(16)_ = 20.230; *p* = 0.210) and after DN treatment (X_(14)_ = 18.449; *p* = 0.187).

Table 5 presents the most commonly reported clinical adverse effects by PTs in the last month, all of which were extremely rare. Nerve paralysis was reported by 3% of respondents, followed by infection (2%) and shingles (1%).

Table 6 presents the most severe adverse effects observed throughout clinical practice, all of which remained extremely rare. Pneumothorax and shock were each observed by 3% of respondents, nerve palsy by 14%, infection by 2%, and hospitalization by 1%. No significant interactions were found between experience in DN and the occurrences of pneumothorax reported during DN treatment (X_(4)_ = 2.676; *p* = 0.613), shock (X_(4)_ = 4.002; *p* = 0.406), nerve palsy (X_(6)_ = 4.280; *p* = 0.639), and infection (X_(2)_ = 2.611; *p* = 0.271).

Table 7 presents the general clinical practices of the PTs. Regarding their regular practice, the majority keep records of adverse effects (79%). An overwhelming majority have never been sued by patients or institutions concerning their DN practices (97%), and 90% have never received a negative review on social media. No significant interactions were found between experience in DN and the records of adverse effects (X_(2)_ = 5.499; *p* = 0.064), although significant interactions were observed in the number of negative social media reviews (X_(2)_ = 11.698; *p* = 0.003), in which it was the more experienced practitioners who reported receiving negative feedback, with 13% indicating they had been negatively reviewed once.

## 4. Discussion

The findings from this study highlight the frequency of mild adverse effects associated with DN treatment, with minor bleeding and slight pain during treatment being the most commonly reported short-term effects reported by the physiotherapists. Post-treatment pain was also reported by over half of the physiotherapists. A retrospective look at the past month revealed similar trends in the frequency of bleeding, bruising, and pain. Interestingly, while prior experience with DN did not significantly influence the reporting of most adverse effects, a relationship was found between experience and the frequency of tingling sensations. Severe adverse effects were rare, with only minor occurrences of pneumothorax, nerve palsy, and infection reported. These results suggest that while mild adverse effects are common, they are generally short-lived and not influenced by previous DN experience, with serious complications remaining infrequent in clinical practice.

Our results revealed that, when reflecting on the past week, physiotherapists reported that the most frequent occurrences were light bleeding, pain during and after treatment, and bruising. Regarding the mild adverse effects reported over the past month, the majority of respondents indicated that bleeding occurred between one and three times, with bruising occurring at a similar frequency. Post-treatment pain was reported to occur one to three times by 44% of respondents, while pain during dry needling was reported at this frequency by 47%. Most respondents reported no instances of symptom exacerbation, drowsiness, weakness, headache, or nausea.

Our results are consistent with a previous study conducted with practitioners in the USA [13] which identified bleeding, bruising, and pain during the procedure as the most common mild adverse effects. Similarly, a survey conducted in Ireland also found that bruising, bleeding, and pain during treatment were the most frequently reported mild adverse effects [14]. These results can be attributed to the fact that DN causes micro-trauma to muscle fibers, fascia, and blood vessels, which can lead to localized bleeding and inflammation. The discomfort or pain experienced during and after treatment is a result of nociceptor (pain receptor) stimulation in the affected tissues, along with the release of inflammatory mediators, such as prostaglandins. Bruising occurs when the needle ruptures small blood vessels (capillaries), allowing blood to leak into the surrounding tissues. These responses are typically mild and short-lived, reflecting the body’s natural healing processes following tissue irritation.

Interestingly, the majority of comparisons between practitioners’ experience with DN and the reported adverse effects showed no significant interactions, suggesting a general lack of a relationship. However, tingling was reported more frequently by practitioners with over 7 years of experience compared to those with 0–2 years or 3–6 years of experience. Additionally, more experienced practitioners (i.e., those with over 7 years of practice) reported 7 to 15 occurrences of drowsiness in the previous month, which are significantly greater numbers of reports than those of the practitioners with 0–2 years or 3–6 years of experience. Experienced practitioners may develop a heightened awareness of the subtleties of treatment responses, leading to more frequent self-reports of sensations like tingling and drowsiness, which may be less likely to be noted or identified by less experienced practitioners. Less experienced practitioners may be more focused on the technical aspects of needle insertion, leading to fewer reports of these sensations. Moreover, it is possible that experienced practitioners, having treated more patients, may be exposed to a wider range of treatment outcomes and side effects, including drowsiness, and thus are more likely to report it.

Regarding severe (or significant) adverse effects, reports from the past month indicate that these events are extremely rare. Nerve paralysis was reported by 3% of respondents, followed by infection and shingles. Over the course of their clinical careers, respondents reported that nerve palsy was the most common severe adverse effect, occurring in 14% of cases. Pneumothorax and shock were each observed in 3% of respondents, while infection was reported by 2% and hospitalization by 1%. No significant relationships were found between DN experience level and the occurrence of adverse effects, in contrast to a previous study [18], which identified that being male, having more than 4 years of experience, and receiving more training were associated with a higher likelihood of major adverse events occurring over the course of one’s career. Despite the rarity of significant adverse effects associated with DN, nerve palsy was the most commonly reported complication among physical therapist respondents. This is likely due to the fact that DN targets muscle trigger points by inserting thin needles into the skin and muscle tissue [1]. While the risk of nerve damage is generally low, certain anatomical factors—such as the proximity of major nerves to muscle trigger points—can increase the likelihood of inadvertent nerve involvement [16]. For example, inserting needles near the sciatic nerve or the brachial plexus, which are more superficially located in certain areas, may elevate the risk of temporary nerve compression or injury [19]. Additionally, factors such as needle depth, technique, and patient characteristics (including body composition or pre-existing neurological conditions) can further influence the likelihood of nerve palsy.

While our study provides valuable insights into the frequency and nature of mild and severe adverse effects associated with DN, some limitations should be considered. One of the limitations is related to the sample size. While the a priori sample size calculation supports the number of practitioners surveyed, we acknowledge that a larger sample would provide greater robustness to the findings. Additionally, the very limited number of non-certified DN practitioners may constrain the balance between those with and without certification. The retrospective design of the survey, relying on practitioners’ recollection of adverse events over the past month or week, introduces the potential for recall bias, which may have affected the accuracy of self-reported data. Moreover, although we asked for the range of DN treatments per week, we did not request the exact numbers. This limitation prevents us from precisely calculating the frequency of occurrences per 1,000 treatments. This study also did not account for other potential confounders, such as patient demographics or specific treatment protocols, which could influence the occurrence of adverse effects. Future research could benefit from prospective studies that track adverse events in real time, reducing the potential for recall bias and providing more accurate data on the frequency and duration of mild and severe complications. Additionally, examining the relationship between patient characteristics, the specific treatment, and adverse outcomes could offer further insight into risk factors for DN-related complications. Although this study has limitations, this study highlights that while mild adverse effects like bleeding, bruising, and pain are common with DN, they are typically short-lived and not significantly influenced by practitioner experience. Severe complications remain rare, although vigilance is necessary when treating areas near major nerves.

In the context of existing research, our study on Polish physiotherapists aligns with findings from other countries, such as the USA [18], Australia [15], and Ireland [14], where the most commonly reported mild adverse effects of DN include bleeding, bruising, and pain. Our study also provides evidence that experience does not appear to play a significant role in the incidence of mild adverse effects, suggesting that selecting certified practitioners offers a safe option without major concerns about their level of experience. Additionally, our findings highlight the importance of the ongoing monitoring of adverse effects to enhance the reporting and understanding of consequences while reinforcing confidence in the safety of DN, given the very low occurrence of severe adverse effects. Future studies should explore procedures and mechanisms for reducing the occurrence of mild effects, such as optimizing DN techniques or incorporating complementary approaches to mitigate these side effects in patients.

## 5. Conclusions

Our study reveals the common occurrence of mild adverse effects associated with DN reported by physiotherapists, such as light bleeding, bruising, and pain during and after treatment. These effects are typically short-lived and not influenced by the practitioner’s level of experience, although more experienced practitioners reported tingling sensations and drowsiness more frequently, possibly due to heightened awareness or greater exposure to diverse treatment outcomes. Severe complications, such as nerve palsy, pneumothorax, and infection, were reported as rare by physiotherapists, with nerve palsy being the most frequently reported severe adverse effect among respondents. Despite the retrospective nature of this study and potential recall bias, the findings suggest that DN is generally a safe procedure, with mild side effects being transient and severe complications occurring infrequently.

## Figures and Tables

**Table 1:** The demographic information of the survey participants.

	>7 Years	0–2 Years	3–6 Years	Total
*n*	15	44	43	102
Sex				
Women	3 (20%)	15 (34%)	14 (33%)	32 (31%)
Men	12 (80%)	29 (66%)	29 (67%)	70 (69%)
Age				
20–25 yo	0 (0%)	11 (25%)	0 (0%)	11 (11%)
26–35 yo	1 (7%)	23 (52%)	20 (47%)	44 (43%)
36–45 yo	7 (47%)	10 (23%)	16 (37%)	33 (32%)
46–55 yo	4 (27%)	0 (0%)	7 (16%)	11 (11%)
56–65 yo	1 (7%)	0 (0%)	0 (0%)	1 (1%)
>66 yo	2 (2%)	0 (0%)	0 (0%)	2 (2%)
Practicing PT				
0–2 years	0 (0%)	14 (32%)	0 (0%)	14 (14%)
3–6 years	0 (0%)	14 (32%)	11 (26%)	25 (25%
7–10 years	1 (7%)	8 (18%)	12 (28%)	21 (21%)
11–15 years	3 (20%)	5 (11%)	11 (26%)	19 (19%)
>16 years	11 (73%)	3 (7%)	9 (21%)	23 (0%)
DN Certification				
No	1 (0%)	5 (11%)	0 (0%)	6 (6%)
Yes	14 (14%)	39 (89%)	43 (100%)	96 (96%)
Weekly DN				
1–10	6 (40%)	30 (68%)	20 (47%)	56 (55%)
11–20	3 (20%)	7 (16%)	8 (19%)	18 (18%)
21–30	1 (7%)	2 (5%)	7 (16%)	10 (10%)
31–40	2 (13%)	2 (5%)	3 (7%)	7 (7%)
41–50	0 (0%)	1 (2%)	0 (0%)	1 (1%)
50–100	1 (7%)	1 (2%)	3 (7%)	5 (5%)
>100	2 (13%)	0 (0%)	2 (5%)	4 (4%)

DN: dry needling; PT: physiotherapy.

**Table 2:** The mild adverse effects reported in the past week.

	>7 Years N = 15	0–2 Years N = 44	3–6 Years N = 43	Total N = 102
Slight pain during treatment	9 (60%)	30 (68%)	30 (70%)	69 (68%)
Slight pain after treatment	5 (33%)	21 (48%)	27 (63%)	53 (52%)
Feeling of weakness	4 (27%)	5 (11%)	10 (23%)	19 (19%)
Light bleeding	7 (47%)	31 (70%)	33 (77%)	71 (70%)
Bruising	2 (13%)	13 (30%)	19 (44%)	34 (33%)
Tingling	4 (27%)	2 (5%)	1 (2%)	7 (7%)
Nausea	1 (7%)	1 (2%)	1 (2%)	3 (3%)
Drowsiness	4 (27%)	4 (9%)	11 (26%)	19 (19%)
Exacerbation of primary pain symptoms	1 (7%)	4 (9%)	2 (5%)	7 (7%)
Headache	0 (0%)	0 (0%)	2 (5%)	2 (2%)

**Table 3:** The adverse effects noticed in the past month.

	>7 Years N = 15	0–2 Years N = 44	3–6 Years N = 43	Total N = 102
Adverse effects as bleeding event				
0	8 (53%)	13 (30%)	11 (26%)	32 (31%)
1–3	5 (33%)	25 (57%)	20 (47%)	50 (49%)
12–15	0 (0%)	1 (2%)	0 (0%)	1 (1%)
15–20	0 (0%)	0 (0%)	1 (2%)	1 (1%)
>20	0 (0%)	1 (2%)	1 (2%)	2 (2%)
4–6	0 (0%)	1 (2%)	6 (14%)	7 (7%)
7–9	2 (13%)	1 (2%)	2 (5%)	5 (5%)
9–12	0 (0%)	1 (2%)	2 (5%)	3 (3%)
Adverse effects as bruising				
0	8 (53%)	20 (45%)	15 (35%)	43 (42%)
1–3	6 (40%)	19 (43%)	20 (47%)	45 (44%)
12–15	0 (0%)	1 (2%)	0 (0%)	1 (1%)
4–6	0 (0%)	2 (5%)	3 (7%)	5 (5%)
7–9	1 (7%)	1 (2%)	2 (5%)	4 (4%)
9–12	0 (0%)	0 (0%)	2 (5%)	2 (2%)
Adverse effects as pain after DN				
0	6 (40%)	16 (36%)	15 (35%)	37 (36%)
1–3	5 (33%)	22 (50%)	20 (47%)	47 (46%)
12–15	0 (0%)	1 (2%)	2 (5%)	3 (3%)
15–20	1 (7%)	1 (2%)	1 (2%)	3 (3%)
>20	1 (7%)	0 (0%)	0 (0%)	1 (1%)
4–6	2 (13%)	1 (2%)	2 (5%)	5 (%)
7–9	0 (0%)	2 (5%)	1 (2%)	3 (3%)
Adverse effects as pain during DN				
0	4 (27%)	12 (27%)	6 (14%)	22 (22%)
1–3	6 (40%)	21 (48%)	21 (49%)	48 (47%)
15–20	0 (0%)	1 (2%)	2 (5%)	3 (3%)
>20	3 (20%)	1 (2%)	2 (5%)	6 (6%)
4–6	1 (7%)	4 (9%)	7 (16%)	12 (12%)
7–9	1 (7%)	2 (5%)	4 (9%)	7 (7%)
9–12	0 (0%)	2 (5%)	1 (2%)	3 (3%)
Adverse effects as exacerbation of primary symptoms				
0	11 (73%)	32 (73%)	33 (77%)	76 (75%)
1–3	2 (13%)	11 (25%)	9 (21%)	22 (22%)
4–6	2 (13%)	0 (0%)	1 (2%)	3 (3%)
Adverse effects as drowsiness				
0	8 (53%)	34 (77%)	26 (60%)	68 (67%)
1–3	3 (20%)	8 (18%)	14 (33%)	25 (25%)
12–15	1 (7%)	0 (0%)	0 (0%)	1 (1%)
4–6	2 (13%)	1 (2%)	0 (0%)	3 (3%)
7–9	1 (7%)	0 (0%)	1 (2%)	2 (2%)
9–12	0 (0%)	0 (0%)	1 (2%)	1 (1%)
Adverse effects as feeling of weakness				
0	8 (53%)	33 (75%)	27 (63%)	68 (67%)
1–3	5 (33%)	9 (20%)	11 (26%)	25 (25%)
15–20	0 (0%)	0 (0%)	1 (2%)	1 (1%)
4–6	0 (0%)	1 (2%)	1 (2%)	2 (2%)
7–9	2 (13%)	0 (0%)	0 (0%)	2 (2%)
9–12	0 (0%)	0 (0%)	2 (5%)	2 (2%)
Adverse effects as headache				
0	11 (73%)	42 (95%)	37 (86%)	90 (88%)
1–3	3 (20%)	1 (2%)	4 (9%)	8 (8%)
9–12	0 (0%)	0 (0%)	1 (2%)	1 (1%)
Adverse effects as nausea				
0	12 (80%)	40 (91%)	36 (84%)	88 (86%)
1–3	2 (13%)	2 (5%)	4 (9%)	8 (8%)
4–6	0 (0%)	0 (0%)	1 (2%)	1 (1%)
7–9	0 (0%)	0 (0%)	1 (2%)	1 (1%)

DN: dry needling.

**Table 4:** Numeric pain rating scale scores* reported in DN procedures.

	>7 Years N = 15	0–2 Years N = 44	3–6 Years N = 43	Total N = 102
During DN procedures				
0	0 (0%)	0 (0%)	3 (7%)	3 (3%)
1–2	4 (27%)	16 (36%)	10 (23%)	30 (29%)
2–3	4 (27%)	6 (14%)	7 (16%)	17 (17%)
3–4	4 (27%)	11 (25%)	12 (28%)	27 (26%)
4–5	1 (7%)	2 (5%)	9 (21%)	12 (12%)
5–6	2 (13%)	5 (11%)	1 (2%)	8 (8%)
6–7	0 (0%)	2 (5%)	0 (0%)	2 (2%)
>6	0 (0%)	0 (0%)	1 (2%)	1 (1%)
7–8	0 (0%)	1 (2%)	0 (0%)	1 (1%)
After DN procedures				
0	2 (13%)	8 (18%)	6 (14%)	16 (16%)
1–2	9 (60%)	21 (48%)	17 (40%)	47 (46%)
2–3	0 (0%)	3 (7%)	9 (21%)	12 (12%)
3–4	3 (20%)	5 (11%)	7 (16%)	15 (15%)
4–5	0 (0%)	3 (7%)	3 (7%)	6 (6%)
5–6	0 (0%)	2 (5%)	0 (0%)	2 (2%)
>6	1 (7%)	0 (0%)	0 (0%)	1 (1%)
7–8	0 (0%)	1 (2%)	0 (0%)	1 (1%)

DN: dry needling; *: these are the average values reported by the physiotherapists based on the average number of participants they treated.

**Table 5:** The clinically severe adverse effects observed in the past month.

	>7 Years N = 15	0–2 Years N = 44	3–6 Years N = 43	Total N = 102
Infection	0 (0%)	2 (5%)	0 (0%)	2 (2%)
Shingles	0 (0%)	1 (2%)	0 (0%)	1 (1%)
Nerve paralysis	0 (0%)	1 (2%)	2 (5%)	3 (3%)

**Table 6:** Clinically significant adverse effects observed throughout clinical practice.

	>7 Years N = 15	0–2 Years N = 44	3–6 Years N = 43	Total N = 102
Pneumothorax				
0	14 (93%)	42 (95%)	40 (93%)	96 (94%)
1	0 (0%)	1 (2%)	0 (0%)	1 (1%)
2	0 (0%)	0 (1%)	2 (5%)	2 (2%)
Shock				
0	13 (87%)	42 (95%)	40 (93%)	95 (93%)
1	0 (0%)	1 (2%)	0 (0%)	1 (1%)
2	1 (7%)	0 (0%)	1 (2%)	2 (2%)
Nerve palsy				
0	12 (80%)	38 (86%)	35 (81%)	85 (83%)
1	2 (13%)	4 (9%)	3 (7%)	9 (9%)
2	0 (0%)	1 (2%)	2 (5%)	3 (3%)
>4	0 (0%)	0 (0%)	2 (5%)	2 (2%)
Infection				
0	14 (93%)	41 (93%)	41 (95%)	96 (94%)
1	0 (0%)	2 (5%)	0 (0%)	2 (2%)
Hospitalization for DN adverse effects				
No	14 (93%)	43 (98%)	41 (95%)	98 (96%)
Pneumothorax	1 (7%)	0 (0%)	0 (0%)	1 (1%)

**Table 7:** General clinical practice.

	>7 Years N = 15	0–2 Years N = 44	3–6 Years N = 43	Total N = 102
Do you keep records of adverse effects?				
No	0 (0%)	7 (16%)	11 (26%)	18 (18%)
Yes	15 (100%)	36 (82%)	30 (70%)	81 (79%)
Have you ever been sued by a patient or professional for the adverse effects of DN practice?				
No	14 (93%)	43 (98%)	42 (98%)	99 (97%)
Yes	0 (0%)	0 (0%)	0 (0%)	0 (0%)
Have you ever been negatively reviewed on social media by a patient related to adverse effects after DN?				
No	13 (87%)	43 (98%)	43 (100%)	99 (97%)
Yes, once	2 (13%)	0 (0%)	0 (0%)	2 (2%)

DN: dry needling.

## Data Availability

Data are available upon request to the corresponding author.

## Supplementary Materials

The following supporting information can be downloaded at https://www.mdpi.com/article/10.3390/jcm13237032/s1.
