# Supplementary material for: Adverse Reactions to Dry Needling Therapy: Insights from Polish Physiotherapy Practice
Source: J Clin Med. 2024 Nov 21;13(23):7032. doi: 10.3390/jcm13237032 (PMC11642314; doi:10.3390/jcm13237032)
Supplement: Supplementary file 1 [file jcm-13-07032-s001.zip › jcm-3328482-supplementary.pdf]

Supplementary file S1. The survey.

Adverse events in using dry needling (DN) in physiotherapy practice.

I kindly ask you to fill out the attached questionnaire. In this way, we want to gain knowledge about adverse events in the use of dry needling (DN) in physiotherapy practice, in order to publish a study on the above-mentioned topic on this basis. The purpose of this study will be to determine the risk and number of adverse events during dry needling therapy. We assure you complete anonymity of your statements. We will use the collected data only in summary statistics.

Thank you in advance for your time.

Adverse events are defined as any bad effect, no matter how small, unintended, and non-therapeutic it is.

1. State your gender

Female

Male

2. State your age

20-25

26-35

36-45

46-55

56-65

66+

3. How many years have you been practicing physiotherapy?

0-2 years

3-6 years

7-10 years

11-15 years

more than 16 years

4. Have you completed a dry needling course?

Yes

No

5. How many years have you been practicing dry needling?

0-2 years

3-6 years

7-10 years

11-15 years

more than 16 years

6. How often do you perform a dry needling treatment in one week? (one injection=one treatment)

1-10

11-20

21-30

31-40

41-50

50-100

100+

7. Which of these clinically insignificant adverse events have you noticed during/after dry needling therapy in the past week?

Light bleeding

Bruising

Slight pain during treatment

Slight pain after treatment

Exacerbation of primary pain symptoms

Drowsiness

Feeling of weakness

Headache

Nausea

Tingling

8. How many times have you noticed an adverse bleeding event in the past month?

0

1-3

4-6

7-9

9-12

12-15

15-20

20+

9. How many times in the past month have you noticed an adverse event in the form of a bruise?

0

1-3

4-6

7-9

9-12

12-15

15-20

20+

10. How many times in the past month have you noticed an adverse event in the form of pain after therapy?

0

1-3

4-6

7-9

9-12

12-15

15-20

20+

11. How many times in the past month have you noticed an adverse event in the form of pain during therapy?

0

1-3

4-6

7-9

9-12

12-15

15-20

20+

Numerical Rating Scale (NRS)

The scale contains 11 degrees of pain intensity - from 0 to 10, where 0 means no pain at all, while 10 means the worst imaginable pain.

12. On the NRS scale, what was the severity of pain during the dry needling procedure?

0

1-2

2-3

3-4

4-5

5-6

6-7

7-8

9-10

13. On the NRS scale, what was the severity of pain after the dry needling procedure?

0

1-2

2-3

3-4

4-5

5-6

6-7

7-8

9-10

14. How many times in the past month have you noticed an adverse event in the form of an exacerbation of primary symptoms?

0

1-3

4-6

7-9

9-12

12-15

15-20

20+

15. How many times in the past month have you noticed an adverse event in the form of drowsiness?

0

1-3

4-6

7-9

9-12

12-15

15-20

20+

16. How many times in the past month have you noticed an adverse event in the form of a feeling of weakness?

0

1-3

4-6

7-9

9-12

12-15

15-20

20+

17. How many times in the past month have you noticed an adverse event in the form of a headache?

0

1-3

4-6

7-9

9-12

12-15

15-20

20+

18. How many times in the past month have you noticed an adverse event in the form of nausea?

0

1-3

4-6

7-9

9-12

12-15

15-20

20+

19. Which of these clinically significant adverse events have you noticed during/after dry needling therapy in the past month?

Pneumothorax

Shock

Nerve paralysis

Infection

None of the above

Other:

20. How many times throughout your clinical practice have you noticed an adverse event during/after dry needling therapy in the form of pneumothorax?

0

1

2

3

4

greater than 4

21. How many times throughout your clinical practice have you noticed an adverse event during/after dry needling therapy in the form of shock?

0

1

2

3

4

more than 4

22. How many times throughout your clinical practice have you noticed an adverse event during/after dry needling therapy in the form of nerve palsy?

0

1

2

3

4

more than 4

23. How many times throughout your clinical practice have you noticed an adverse event during/after dry needling therapy in the form of an infection?

0

1

2

3

4

more than 4

24. Do you keep a record of adverse events (e.g., an entry in the patient's chart or an entry that such an event occurred at a particular visit)

Yes

No

25. Did any of your patients have to be hospitalized for adverse events after a dry needling procedure?

Yes, if yes, write below how many days the hospitalization lasted and what the effect was

No

Other:

26. Have you ever been sued by a patient or professional control institution for adverse events after a dry needling procedure?

Yes, if yes describe below what the outcome of the procedure was

No

Other:

27. Have you ever been negatively reviewed on social media by a patient in connection with adverse events after a dry needling procedure?

Yes, if yes write below how many times

No

Other:
